# Data Quality–Driven Improvement in Health Care: Systematic Literature Review

**DOI:** 10.2196/57615

**Published:** 2024-08-22

**Authors:** Anthony Lighterness, Michael Adcock, Lauren Abigail Scanlon, Gareth Price

**Affiliations:** 1 Clinical Outcomes and Data Unit The Christie NHS Foundation Trust Manchester United Kingdom; 2 Radiotherapy Related Research Group University of Manchester Manchester United Kingdom

**Keywords:** real-world data, data quality, quality improvement, systematic literature review, PRISMA

## Abstract

**Background:**

The promise of real-world evidence and the learning health care system primarily depends on access to high-quality data. Despite widespread awareness of the prevalence and potential impacts of poor data quality (DQ), best practices for its assessment and improvement are unknown.

**Objective:**

This review aims to investigate how existing research studies define, assess, and improve the quality of structured real-world health care data.

**Methods:**

A systematic literature search of studies in the English language was implemented in the Embase and PubMed databases to select studies that specifically aimed to measure and improve the quality of structured real-world data within any clinical setting. The time frame for the analysis was from January 1945 to June 2023. We standardized DQ concepts according to the Data Management Association (DAMA) DQ framework to enable comparison between studies. After screening and filtering by 2 independent authors, we identified 39 relevant articles reporting DQ improvement initiatives.

**Results:**

The studies were characterized by considerable heterogeneity in settings and approaches to DQ assessment and improvement. Affiliated institutions were from 18 different countries and 18 different health domains. DQ assessment methods were largely manual and targeted completeness and 1 other DQ dimension. Use of DQ frameworks was limited to the Weiskopf and Weng (3/6, 50%) or Kahn harmonized model (3/6, 50%). Use of standardized methodologies to design and implement quality improvement was lacking, but mainly included plan-do-study-act (PDSA) or define-measure-analyze-improve-control (DMAIC) cycles. Most studies reported DQ improvements using multiple interventions, which included either DQ reporting and personalized feedback (24/39, 61%), IT-related solutions (21/39, 54%), training (17/39, 44%), improvements in workflows (5/39, 13%), or data cleaning (3/39, 8%). Most studies reported improvements in DQ through a combination of these interventions. Statistical methods were used to determine significance of treatment effect (22/39, 56% times), but only 1 study implemented a randomized controlled study design. Variability in study designs, approaches to delivering interventions, and reporting DQ changes hindered a robust meta-analysis of treatment effects.

**Conclusions:**

There is an urgent need for standardized guidelines in DQ improvement research to enable comparison and effective synthesis of lessons learned. Frameworks such as PDSA learning cycles and the DAMA DQ framework can facilitate this unmet need. In addition, DQ improvement studies can also benefit from prioritizing root cause analysis of DQ issues to ensure the most appropriate intervention is implemented, thereby ensuring long-term, sustainable improvement. Despite the rise in DQ improvement studies in the last decade, significant heterogeneity in methodologies and reporting remains a challenge. Adopting standardized frameworks for DQ assessment, analysis, and improvement can enhance the effectiveness, comparability, and generalizability of DQ improvement initiatives.

## Introduction

### Background

The landscape of health care, improvement science, and digital technologies increasingly hinges on real-world data (RWD) to improve patient care and outcomes [1,2]. RWD encompasses a vast and dynamic collection of health-related information generated by means of routine clinical care from a diverse range of sources, such as electronic health records (EHRs), electronic medical records (EMRs), hospital information systems (HISs), picture archiving and communication systems (PACSs), national registries, claims data, and wearable devices [2-4]. Despite their long history, the adoption and use of EHRs have become widespread only during the last decade [4,5]. EHRs and EMRs are often used interchangeably in the literature discussing health-related RWD, where some suggest that EMRs are a subset of EHRs [6], but the prominence of EHRs have positioned them as a primary source of RWD due to the comprehensive spectrum of patient information, including genetic testing, treatment modalities, and clinical outcomes [7,8]. To reflect this primary focus, the term *EHR-RWD* will be used throughout this review to denote RWD derived from EHRs.

Real-world evidence (RWE) generated from EHR-RWD holds unprecedented potential to bridge the unmet gaps that exist between controlled clinical trial studies and the complexities of health care delivery in the real world [1,2,7,9]. While randomized control trial studies remain the “gold standard” for assessing the efficacy of new interventions, the essence of RWE lies in its potential to reflect the diversity, heterogeneity, and nuances of patient populations and care settings, thus enabling a more holistic understanding of health outcomes and interventions [7,10]. Data and RWE studies can support the life cycle of drug development, clinical and regulatory decision-making, and health technology assessment [3,8,11]. Moreover, RWE underpins the vision of the learning health care system (LHS), which is a paradigm built upon the cycle of continuous learning to achieve personalized medicine [12]. The transformative potential of RWE, however, hinges on a pivotal caveat—high-quality data.

Despite its potential, EHR-RWD, and by extension RWE, grapple with formidable barriers, and chief among these is data quality (DQ) [1,2,9,10,13,14]. The need for high-quality data was exemplified during the COVID-19 pandemic, when EHR-RWD was critical for research and planning [15]. Regulatory authorities, such as the Food and Drug Administration (FDA), European Medicines Agency (EMA), National Institute for Health and Care Excellence (NICE), and Medicines and Healthcare Products Regulatory Agency (MHRA), recommend the reporting of DQ metrics and dimensions to provide additional context to real-world study outcomes, thus serving as the foundation for trustworthy RWE [3,14,16-19]. These guidelines mostly promote ad hoc DQ assessment and reporting, with the exception that EMA briefly notes the importance of assessing DQ as close as possible to the moment of data capture to help with collection errors [3,16-18]. While ad hoc DQ measurement and reporting can increase transparency and awareness of the limitations of real-world study outcomes, understanding the causes of poor data capture is needed for long-term, sustainable improvement in DQ and, subsequently, the impact of RWE to support decision-making in health care.

In this study, we aimed to evaluate the robustness of studies seeking to improve the quality of structured EHR-derived data. Incorporating quality improvement best practices, we aimed to assess how studies measure DQ, identify which interventions are implemented, and summarize the outcomes to understand which interventions are useful to improve DQ.

### DQ Theory

The quality of data describes if the data meet the expectations of a data consumer and, therefore, if they are fit for purpose [20]. This expected behavior can be documented and understood using metadata, that is, additional data that provide meaning and context by describing how the data they are associated with should have been captured, defined, structured, and represented [20]. Metadata, in turn, can inform the design of quantifiable metrics that measure the compliance of data against a set of relevant business rules and constraints during DQ assessment [21]. DQ metrics, which can act as the method by which to measure the respective DQ dimensions, can serve a crucial purpose in understanding areas needing improvement.

Scholars have proposed various multidimensional frameworks for comprehensive DQ assessment. While there may be disagreements on semantic choices and definitions across these frameworks, certain DQ concepts are consistently studied and well represented in the literature, popular frameworks, and DQ profiling software [22]. Despite the challenge, some common concepts can be mapped across frameworks, as exemplified by the 6 core dimensions defined by the Data Management Association (DAMA): completeness, validity, consistency, uniqueness, timeliness, and accuracy (Textbox 1) [20].

Textbox 1 outlines the 6 fundamental DQ dimensions defined by DAMA to enable standardization and comparability of DQ concepts defined, assessed, and improved in the included DQ improvement literature. These dimensions are essential for assessing and improving the quality of RWD.

In an exploration of the DQ literature, we found 10 other reviews that summarized the most frequently represented DQ dimensions across DQ studies, software, and theoretical frameworks [19,21,23-30]. All 10 reviews demonstrated the concept of completeness to be well represented. Of these, 4 reviews agreed that data accuracy and consistency were popular DQ concepts [23,24,26,28]. Timeliness and validity were said to be popular by 3 different reviews, and uniqueness was only raised in the study by Gordon et al [29].

**Core dimensions of data quality as defined by the Data Management Association.**

**Data quality dimension and description**

- Completeness: the presence of the expected data
- Uniqueness: uniqueness of records where duplication is not expected
- Timeliness: a measure of data freshness
- Consistency: a check of consistency between multiple sources of the same data elements
- Validity: the validity of data against data standards or plausible values, ranges, or patterns
- Accuracy: a check of consistency of source data against a reference
  gold standard

Completeness, also known as missingness, is often reported to be popular among DQ frameworks, tooling, and studies [22-27,29,30]. In general, it refers to the degree to which all required or expected data values or records are present [20]. The most common method to measure completeness consists of counting records with blank, unknown, empty, “NULL,” or “NaN” values, though variations may include a measurement of data availability [22,25,26]. Reviews by Weiskopf and Weng [22] and Syed et al [28] also found variations of data completeness assessment involving triangulation of multiple sources to create a gold standard. However, this approach risks assuming the accuracy of available data. Previous work demonstrate that missing data can lower statistical power of research outcomes and lead to biased assumptions with improper use of imputation methods [10,31,32].

Data accuracy is also well studied in DQ literature [22,25,28]. The accuracy dimension measures the extent to which data reflect the truth of events and conform to their actual value [20,22,25,28]. Other terms used to describe accuracy include error, correctness, integrity, trustworthiness, reliability, and validity [22,28]. The most common method to assess accuracy in health care involves the comparison of EHR data to a reference gold standard, which may include paper records, manual data reviews, triangulation of data from multiple sources, or interviews with patients [22]. Measurement of data accuracy can identify issues such as lack of specificity or precision [33]. Previous work found that code precision can be related to staff training or use of multiple EHR systems [33-36]. As Cook et al [37] noted in a review of DQ issues affecting social determinants data, imprecise codified data may affect minority groups disproportionately, which in turn may affect secondary research outcomes.

Various terms and definitions for validity exist in the literature [22,28]. It generally describes the conformance of data to expected value ranges, patterns, formats, general medical knowledge, or data standards as set by local or external authorities [22,26,28,38]. Validity is also termed plausibility, conformance, and integrity and can also be separated into internal and external validity or be incorporated with other data elements such as temporal validity [22,38]. As EHR-derived data contain large volumes of categorical data, such as patient demographic, diagnostic, and treatment-related information, validity constraints are needed to identify areas needing standardization [39,40]. Data standardization has been shown to correlate positively to data sharing capabilities and emergency care [41]. However, improper design of standardized data entry user interfaces, such as the use of excessively long drop-down lists for diagnostic codes, can also increase cognitive demand, lower workflow efficiency, and correlate to clinician burnout [42].

Timeliness refers to several time-related characteristics of data and is, therefore, also termed currency, recency, or freshness [22,38]. For example, time-related data items can measure how closely the recorded information corresponds to the actual event. Factors affecting timely capture of EHR data include workflow inefficiencies, documentation burden, limited access to hardware, and interruptions [43-48]. Batch processing of data long after the event may indicate a lack of timeliness and affects other DQ dimensions such as completeness, accuracy, and validity [28,44,46,48,49].

Consistency, otherwise known as concordance, describes the agreement of similar data elements between multiple sources [20,22]. The existence of multiple data capture systems and RWD sources can give rise to inconsistent data for a given patient, and in the absence of a defined gold standard, the consistency dimension can identify potentially erroneous data [10,50]. Botsis et al [36] identified multiple inconsistencies during a DQ analysis of a cohort of patients with pancreatic cancer stored in the Columbia University Medical Center’s EHR data warehouse. These included pancreatitis recorded as chronic in pathology reports but acute in clinical notes and patients with diabetes receiving both codes for type 1 and type 2 in the same EHR source [36]. von Lucadou et al [51] found similar discrepancies when comparing data items between different systems, adding that inconsistencies may be caused by individual documentation habits. Measurement of data consistency highlights potential duplication and redundancy between different EHR sources and within the same EHR system and can thus help improve data capture or data engineering workflows.

The uniqueness dimension identifies where duplication of objects, events, or values are not expected [10,26]. Duplication of patient EHRs frequently occur when disparate data flows that contain overlapping objects are combined [10,38]. Similar to consistency, the uniqueness dimension can identify and resolve redundant and inefficient workflows and processes [52]. This is particularly relevant given that 60% to 90% of clinicians routinely copy and paste data between systems [53]. The “copy and paste” phenomenon is pervasive in health care and is known to promote inconsistencies, propagate errors, and contribute to documentation burden and clinician fatigue [42,48,53]. As such, the uniqueness dimension is related to consistency and accuracy.

### DQ Tooling

DQ measurement involves the process by which defective values are identified and labeled through the application of business rules or automated tooling [21]. The subsequent analysis of DQ results can then be aggregated, analyzed, and summarized, providing key insights for improvement. Tools to support these activities are widely reported and well studied [21,27,29,30,54,55].

While the availability of DQ tools is abundant, the literature reveals a considerable gap in the effective support for DQ improvement efforts, particularly in the realm of health care. Evaluations of DQ profiling software in the studies by Ehrlinger and Wöß [30], Gordon et al [29], and Ozonze et al [21] highlight limitations in the range of DQ metrics offered for assessment, interoperability issues, and complex configuration requirements.

Root cause analysis, a pivotal aspect of DQ management, is also notably lacking in demonstrations within this landscape. Eden et al [48] demonstrated the utility of the Odigos framework in qualitative root cause analysis, which classifies DQ issues that emanate from the material world, such as digital infrastructure or access to hardware; personal world, that is, staff behaviors; and societal world, that is, job roles and social norms. The legal and technical implications associated with data cleaning, as opposed to addressing the root causes of poor data, underscore potential risks to patient safety [56-58]. Consequently, the overarching trend in DQ tool development leans toward prioritizing technical features, leaving a noticeable gap in the demonstration of their utility in the prevention and improvement of poor-quality data capture in real-world health settings.

### Quality Improvement

Quality improvement describes the use of systematic continuous approaches to create positive changes in an area of need [59]. Various structured, iterative learning frameworks, such as plan-do-study-act (PDSA), total data quality management (TDQM), define-measure-analyze-improve-control (DMAIC), and the LHS, exist [12,60-63]. Lacking a universally agreed-upon model, each methodology focuses on enhancing different areas, ranging from service evaluation to treatment standards [59].

Developed from the earlier plan-do-check-act cycle by Deming [64], the PDSA cycle enhances the traditional model by prioritizing the “study” stage—a deeper analysis rather than a simple check [65]. This adaptation roots the PDSA cycle firmly in the scientific method, encouraging a disciplined approach to testing and monitoring changes over time [65]. Its flexible and qualitative nature makes it particularly suitable for health care settings where adaptability to complex and variable processes is crucial.

Proposed by Wang [61] in the late 1990s, TDQM adapts traditional total quality management principles specifically to data management, highlighting the importance of data as a key asset or product. In health care, where decision-making increasingly relies on accurate and timely data, TDQM offers a robust framework to ensure the integrity and usability of data. This focus on DQ management is critical as health care systems integrate more digital processes and data-driven decision-making frameworks. TDQM adapts the PDSA planning stage to specifically target the improvement of DQ [61].

A product of Motorola engineers in the 1980s, DMAIC provides a structured, data-driven quality improvement methodology [61]. Unlike the more qualitative PDSA, DMAIC emphasizes quantifiable metrics and statistical analysis to identify and mitigate variations in processes. This makes DMAIC highly suitable for health care areas requiring high levels of measurement precision and control, such as clinical laboratories or any clinical process where outcomes need to meet high standards of care.

While all 3 methodologies share a structured, iterative approach and a reliance on empirical data to drive improvements, they cater to different needs within the health care sector. PDSA’s qualitative and flexible nature is best suited for areas requiring rapid change and adaptability. In contrast, DMAIC’s rigorous, statistical approach fits environments where precision and control are paramount. TDQM’s specific focus on DQ fills a critical niche in ensuring the reliability of health care data systems. What remains unknown is their implementations in the real world. In a review of PDSA cycles aimed at improving treatment standards, Taylor et al [60] demonstrated that <20% of implementations comply with the core features including running multiple iterative learning cycles, the notion of small-scale change, and the use of quantitative data at monthly or more frequent intervals to inform progression of cycles. The stages of each methodology are listed and described in Table 1.

Table 1 presents a comparison of the stages involved in 3 iterative learning frameworks present in health quality improvement and other related literature. The comparison aims to understand common themes in quality improvement methodology and how these can be applied to DQ improvement.

**Table 1:** Detailed comparison of 3 QI^a^ frameworks for iterative learning.

Framework and stage			Description
**PDSA^b^ [60]**			
	Plan	Identify a change hypothesis and plan a small test.	
	Do	Conduct a study plan with the collection of data.	
	Study	Analyze and interpret the results.	
	Act	Adapt the change based on feedback and plan the next iteration.	
**TDQM^c^ [61]**			
	Define	Define target data requirements and DQ^d^ dimensions.	
	Measure	Create metrics to evaluate these dimensions.	
	Analyze	Investigate root causes for DQ issues.	
	Improve	Identify key areas for improvement based on DQ root cause analysis.	
**DMAIC^e^ [63]**			
	Define	Define project scope and objectives.	
	Measure	Identify and measure baseline service indicators.	
	Act	Analyze baseline metrics and identify causes of errors.	
	Improve	Implement changes to reduce or remove root causes of defects.	
	Control	Put mechanisms in place to ensure sustained improvement.	

^a^QI: quality improvement.

^b^PDSA: plan-do-study-act.

^c^TDQM: total data quality management.

^d^DQ: data quality.

^e^DMAIC: define-measure-analyze-improve-control.

### Objectives

The rapidly growing body of DQ publications and software tools indicate that this field has gained significant traction, and recent publications illustrate that there is no shortage of DQ concepts, frameworks, and tools [21,24,28-30,54,55,66,67]. While these surveys already provide comprehensive theoretical and functional evaluations on existing DQ concepts and tools for definition and measurement, this represents only the early stages of a bigger picture in DQ improvement and management. Our aim was to evaluate the robustness of studies seeking to use DQ measurement as part of DQ improvement initiatives, focusing on improving the quality of structured EHR-derived data.

Brouwer et al [67], Wiebe et al [68], and Lemma et al [69] have previously published studies on DQ-driven improvement in health care. These studies are compared in Table 2. Wiebe et al [68] included 24 studies aiming to improve EHR documents such as operative reports or discharge summaries. The authors reported that heterogeneity in tools or metrics used to measure the quality of unstructured clinical notes made it difficult to evaluate outcomes. However, 8 included studies used an ad hoc questionnaire and 1 used the validated Physician Documentation Quality Instrument (PDQI-9) tool. Although unstructured notes in health care is a pervasive and ubiquitous source of important patient information, this scope limits the exploration of semiautomated or automated DQ assessment tools or methods [68].

Table 2 aims to compare various literature reviews focused on DQ improvement, identifying current knowledge gaps and evaluating the existing body of research. The goal is to understand current progress and unmet needs.

Brouwer et al [67] evaluated studies published up to 2005 and limited to a general practice setting. With digital health care technology and culture evolving rapidly, a more recent and broader evaluation is needed. Lemma et al [69] focused on low- and middle-income countries, where initiatives generally targeted broader and less-specific DQ improvement compared to high-income countries or technologically advanced institutions. The review expands on these works by evaluating contemporary DQ improvement studies targeting structured her-derived RWD agnostic of health care settings. Our evaluation is guided by quality improvement best practices to understand how studies measure and seek to improve DQ dimensions as defined by the well-recognized DAMA framework [20]. Specifically, we addressed the following three questions:

1. How do quality improvement studies define and measure the quality of data?
2. What interventions are being implemented to improve the quality of RWD?
3. What are the outcomes reported?

**Table 2:** Comparison of literature reviews evaluating DQ^a^ improvement studies.

Comparison	Review paper		
	Brouwer et al [67]	Wiebe et al [68]	Lemma et al [69]
Period covered	Up to 2005	2004-2016	2008-2020
Number of studies	12	24	20
Structured RWD^b^	✓^c^		✓
Completedness	✓	✓	✓
Accuracy	✓	✓	✓
Timeliness		✓	✓
Consistency			✓
Validity			
Uniqueness			
QI^d^ framework			

^a^DQ: data quality.

^b^RWD: real-world data.

^c^Did evaluate.

^d^QI: quality improvement.

## Methods

### Search Strategy and Information Sources

In this review, studies seeking to improve the quality of structured EHR data were examined using the PRISMA (Preferred Reporting Items for Systematic Reviews and Meta-Analyses) guidelines (refer to Multimedia Appendix 1) [70]. The Population - Intervention - Comparison - Outcome - Context framework was used to identify relevant keywords and Medical Subject Headings (MeSH) terms [71,72]. These were combined using Boolean operators to create strategic search queries, which were then used to search the Ovid MEDLINE and PubMed databases for articles published from 1945 to July 2023 (refer to Multimedia Appendix 2 for more information). Additional relevant papers were identified from other publications and manual searches through Google Scholar.

### Literature Selection Process

**Figure 1:**
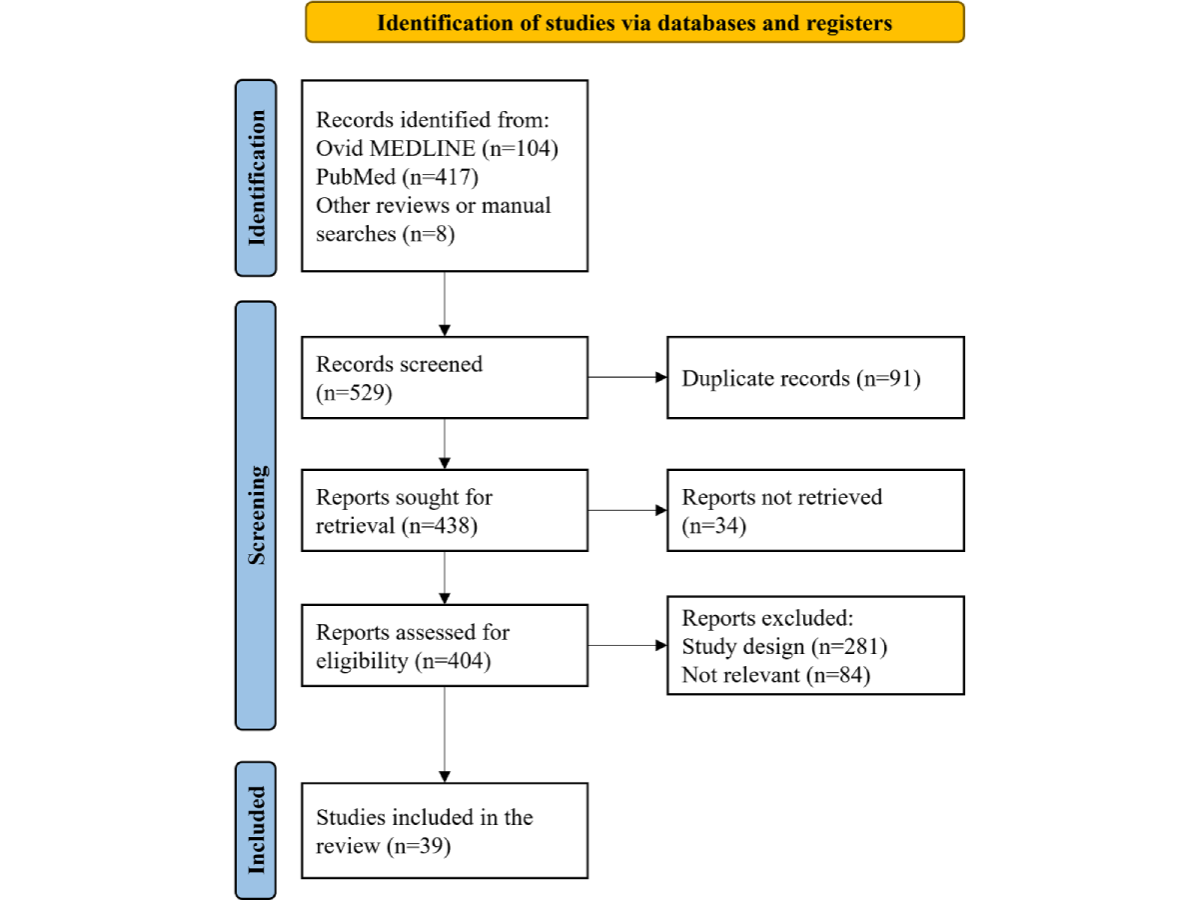
PRISMA (Preferred Reporting Items for Systematic Reviews and Meta-Analyses) flowchart for study selection to evaluate the current literature targeting assessment, analysis, and improvement of the quality of structured real-world data in health care. This PRISMA flowchart illustrates the selection process for reports included in the review, detailing the steps of inclusion and exclusion needed to accurately achieve the intended scope of the study.

The Ovid MEDLINE and PubMed search results were downloaded as a research information systems file and PubMed text file, respectively. These were then imported into the Mendeley reference manager (Mendeley Ltd) and Rayyan (Rayyan Systems Inc) software for iterative analysis [73]. The Rayyan web app was used to streamline the selection process. Articles were selected based on the following criteria: (1) they describe a DQ assessment or measurement process, (2) they focus on data from an EHR or EMR system, and (3) they involve an intervention aimed at improving DQ over time. The search strategies and article selection process were performed independently by 2 reviewers: AL and MA. Excluded articles were nonempirical studies, improvement studies focusing on quality of care or treatment standards instead of quality of data, studies targeting semistructured or unstructured data or data not captured by an RWD source such as an EHR or EMR system, and studies without an intervention seeking to improve DQ. Table 3 summarizes the key inclusion and exclusion criteria for paper selection. In total, 39 studies were included in the review, as presented by the PRISMA flow diagram for RWD (Figure 1).

Table 3 details the specific criteria used to include or exclude studies in the evaluation of current methods for assessing and improving the quality of structured health RWD. These criteria help to systematically assess the landscape of DQ assessment and improvement strategies.

**Table 3:** Inclusion and exclusion criteria to evaluate the current landscape of DQ^a^ assessment and improvement approaches.

Category	Inclusion criteria	Exclusion criteria
Article type	Empirical, original, or review articles where tools, frameworks, or interventions seek to measure and improve DQ	Nonempirical studies, thesis papers, and non–peer-reviewed publications
Language	Published in English	Articles not published in English
Access	Peer-reviewed and open access articles	Papers that are not free to access
Primary target for quality improvement	Studies that primarily aim to improve the quality of data	Studies that primarily target improvement of treatment standards, standard of care, and clinical workflows without a DQ focus
Study population	Studies targeting structured, tabular data	Studies targeting semistructured or unstructured data
Data source	Data from RWD^b^ sources such as EHR^c^, EMR^d^, PACS^e^, or HIS^f^-like systems	Data generated by clinical trial studies
DQ assessment and reporting	Studies that describe a DQ assessment, quantification, or measurement process and implemented an intervention seeking to improve DQ over time	Studies that focus on DQ tool development without demonstration of measurement or improvement of DQ over time
Location or health context	No criteria applied	No criteria applied
Time frame	Studies published since 1945	Studies published before 1945

^a^DQ: data quality.

^b^RWD: real-world data.

^c^EHR: electronic health record.

^d^EMR: electronic medical record.

^e^PACS: picture archiving and communication system.

^f^HIS: hospital information system.

### Data Extraction and Synthesis

Following paper selection, we defined a set of data elements essential for addressing the defined research questions. Subsequently, 2 authors (AL and MA) independently extracted and documented this information from each study. The collected data were then cross-checked for notable discrepancies, and any disparities were resolved through consensus. Five key sections of information were extracted: (1) study characteristics (eg, year of publication and health domain), (2) study plans outlined (eg, descriptions of target data and relevant metadata to plan DQ assessment), (3) DQ assessment (eg, methods and dimensions), (4) interventions (eg, which interventions were implemented seeking to improve DQ?), and (5) outcomes (eg, how are results reported?).

## Results

### Overview of the Study Characteristics

We identified 39 studies describing DQ improvement initiatives in health care that targeted structured RWD sources [74-112]. These are listed in Tables 4 and 5. Most were published between 2009 and 2022 (37/39, 95%), with 2 published in 2002. Studies were affiliated with institutions in the United States (15/39, 38%), followed by Kenya (4/39, 10%), Australia (2/39, 5%), or 3% (1/39) each with 15 other countries. We classified the affiliated institutions into different levels of health care, including primary (11/39, 28%), secondary (10/39, 26%), tertiary (15/39, 38%), and community (3/39, 8%). The primary domains of health care were general practice (4/39, 10%), HIV care (4/39, 10%), intensive care (4/39, 10%), tropical medicine (4/39, 10%), oncology (3/39, 8%), surgery (3/39, 8%), or 3% (1/39) each from 12 other domains.

Table 4 lists the 6 DQ dimensions defined by DAMA, as described, assessed, and/or improved in all included studied. It highlights the framework’s role in standardizing and comparing DQ concepts within the reviewed literature.

DQ improvement studies targeted RWD sources that were generated by varying numbers of institutions. Target data were generated by either a single organization (9/39, 23%) or multiple different sites, which ranged from 2 to 10 (11/39, 28%), 11 to 50 (9/39, 23%), or >51 (9/39, 23%) different organizations. A variety of terminology were used to describe the source systems, including EMR (12/39, 31%), national registries or databases (8/39, 21%), EHR (7/39, 18%), HIS (7/39, 18%), clinical information networks (6/39, 15%), PACS (1/39, 3%), or claims data (1/39, 3%).

**Table 4:** Standardized data quality (DQ) dimensions defined by DAMA^a^ across the included studies.

ID	Study, year	Completeness (n=31)	Validity (n=12)	Uniqueness (n=4)	Consistency (n=11)	Timeliness (n=6)	Accuracy (n=12)
1	de Lusignan et al [109], 2002	✓^b^	✓		✓	✓	
2	Wallace et al [108], 2002	✓					✓
3	Nassaralla et al [105], 2009	✓					✓
4	Amoroso et al [112], 2010						
5	Griever et al [107], 2011	✓					✓
6	Ahlbrandt et al [96], 2012						✓
7	Mphatswe et al [110], 2012	✓					✓
8	Rahbar et al [106], 2013	✓	✓		✓		✓
9	Knight et al [101], 2014	✓				✓	
10	Siegel et al [100], 2014	✓					
11	Benard et al [111], 2015	✓					
12	Genet et al [104], 2015						
13	Haskew et al [99], 2015	✓					
14	Smith et al [95], 2015	✓		✓			
15	Soto et al [102], 2015	✓					
16	Taggart et al [98], 2015	✓	✓	✓	✓		
17	Ewing et al [103], 2016						✓
18	Ma et al [77], 2016	✓	✓		✓	✓	
19	Tuti et al [97], 2016	✓					
20	Qin et al [93], 2017	✓					
21	Edgerton et al [92], 2018	✓					
22	Miyoshi et al [89], 2018	✓	✓	✓			
23	Muthee et al [94], 2018	✓			✓		
24	Qualls et al [90], 2018	✓	✓				
25	Daniel et al [88], 2019	✓	✓	✓	✓		
26	Bhattacharya et al [82], 2020	✓			✓	✓	✓
27	Dean et al [75], 2020					✓	✓
28	Koo et al [81], 2020						
29	Moomba et al [85], 2020	✓	✓				
30	Ng et al [78], 2020	✓					✓
31	Njugunaet al [91], 2020		✓				
32	Sinaiko et al [87], 2020	✓					
33	Larrow et al [74], 2020	✓					✓
34	Manesen et al [83], 2021	✓			✓		
35	Olagundoye et al [84], 2021						✓
36	Tizifa et al [86], 2021	✓			✓		
37	Kiogou et al [80], 2022	✓	✓		✓	✓	
38	Pfaff et al [79], 2022	✓	✓		✓		
39	Tuti et al [76], 2022	✓	✓				

^a^DAMA: Data Management Association.

^b^Did assess and target the DQ dimension for improvement.

**Table 5:** Themes of interventions for DQ^a^ improvement in included studies.

ID	Study, year	IT or technical (n=21)	Training (n=17)	DQ report and feedback (n=24)	Workflow (n=5)	Cleaning (n=3)
1	de Lusignan et al [109], 2002			✓^b^		
2	Wallace et al [108], 2002	✓	✓			
3	Nassaralla et al [105], 2009		✓	✓		
4	Amoroso et al [112], 2010	✓	✓	✓		
5	Griever et al [107], 2011		✓		✓	
6	Ahlbrandt et al [96], 2012	✓	✓			
7	Mphatswe et al [110], 2012		✓	✓		
8	Rahbar et al [106], 2013			✓		✓
9	Knight et al [101], 2014	✓	✓	✓		
10	Siegel et al [100], 2014			✓		
11	Benard et al [111], 2015	✓				
12	Genet et al [104], 2015				✓	
13	Haskew et al [99], 2015	✓		✓		
14	Smith et al [95], 2015	✓				
15	Soto et al [102], 2015	✓	✓	✓		
16	Taggart et al [98], 2015			✓		
17	Ewing et al [103], 2016	✓	✓			
18	Ma et al [77], 2016	✓				
19	Tuti et al [97], 2016		✓	✓		
20	Qin et al [93], 2017	✓			✓	
21	Edgerton et al [92], 2018					
22	Miyoshi et al [89], 2018	✓				
23	Muthee et al [94], 2018	✓		✓		
24	Qualls et al [90], 2018			✓		
25	Daniel et al [88], 2019	✓	✓	✓		
26	Bhattacharya et al [82], 2020		✓	✓		
27	Dean et al [75], 2020	✓				
28	Koo et al [81], 2020	✓	✓	✓		
29	Moomba et al [85], 2020				✓	
30	Ng et al [78], 2020	✓	✓	✓		✓
31	Njugunaet al [91], 2020			✓		
32	Sinaiko et al [87], 2020			✓		
33	Larrow et al [74], 2020	✓	✓		✓	
34	Manesen et al [83], 2021	✓		✓		
35	Olagundoye et al [84], 2021	✓	✓	✓		
36	Tizifa et al [86], 2021	✓	✓	✓		
37	Kiogou et al [80], 2022					✓
38	Pfaff et al [79], 2022			✓		
39	Tuti et al [76], 2022			✓		

^a^DQ: data quality.

^b^Did implement.

### DQ Assessment Methods

We found various approaches to DQ assessment. The duration of studies ranged from 1 month to 9 years, as did the frequency of DQ assessment. Most studies measured and reported DQ before and after the intervention (38/39, 97%) at varying intervals, including a single before and after comparison (19/39, 49%), yearly (4/39, 10%), quarterly (2/39, 5%), monthly (11/39, 28%), fortnightly (1/39, 3%), weekly (1/39, 3%), or specified data cycles (1/39, 3%). DQ assessment was achieved using manual (15/39, 38%), automated (3/39, 8%), or semiautomatic (13/39, 33%) methods, whereas some methods lacked sufficient description (6/39, 15%) to be classified. Semiautomated methods for DQ assessment mostly involved the scheduling of ad hoc, manually curated programmatic scripts using either R, SAS, or structured query language (SQL) programming languages [76,85,88,90,91,96-99,101,106-108]. Furthermore, 3 studies applied automated methods that used the World Health Organization (WHO) DQ assessment tool and the Open Data Kit [82,91,94] but did not explicitly describe how this was implemented.

The approaches to defining and assessing DQ dimensions varied. We found that 6 studies explicitly referenced 1 of 2 DQ frameworks, including Weiskopf and Weng [22] (3/39, 8%) and Kahn et al [38] (3/39, 8%) [79,80,82,88,90,94]. To enable comparison between studies, the DQ metrics and dimensions reported were extracted and classified according to the DAMA DQ framework. Some DQ concepts lacked sufficient detail to allow classification (eg, studies reporting “error rate,” “wrong data,” and “percentages of correctly coded” [93,96,112]). These were classified as “unclear” (8/39, 21%). DQ improvement studies assessed the dimensions of completeness (31/39, 79%), accuracy (12/39, 31%), validity (12/39, 31%), consistency (11/39, 28%), timeliness (6/39, 15%), and uniqueness (4/39, 10%). The number of DQ dimensions targeted per study were 1 (13/39, 33%); 2 (14/39, 36%); 3 (2/39, 5%); or 4 (7/39, 18%).

Studies reported inconsistent terminology and definitions for DQ dimensions. For example, although the completeness dimension was generally assessed as the presence or absence of expected data, variations included the proportion of linkage of records between systems [109], use of a gold standard to identify missing patients [92], or overlapping completeness with other dimensions such as validity [76] or accuracy [108]. Validity, also termed conformance or plausibility, was targeted by 12 studies. Of these 39 studies, 6 (15%) used data standards such as WHO International Classification for Disease (ICD) version 9 or 10 [77,85,89,90], SNOMED [77,79,89], Health Language 7 (HL7) [77,88,89], or RxNorm [90]. Others assessed validity by defining business rules that incorporated expected values, formats, or ranges based on local or general medical knowledge [85,89,90,98]. Studies occasionally equated validity with accuracy or correctness [76,89,98].

Of the 12 studies that targeted accuracy, 8 (67%) reported the development and/or use of a gold standard for reference. However, varying definitions for what studies deemed to be a “gold standard” were provided. This included paper charts [93,108]; national data [91,110]; a manually curated data set [84,106]; or manual validation by a trained, expert clinical coder [103]. For example, the “gold standard” in the study by Rahbar et al [106] included 30 patient records that were manually abstracted by a team of experts that included a vascular neurologist clinician before comparing to national stroke registry records. In another study by Ahlbrandt et al [96], the gold standard was described as “the documented and encoded (using OPS [Operationen und Prozedurenschlüssel] Classification, the German modification of ICPM [International Classification of Procedures in Medicine]) surgical procedure,” which could be interpreted either as a data standard or patient data in electronic or paper form.

Sometimes DQ dimensions were subsumed by another. For example, data could only be deemed accurate when they were both complete and correct across multiple data elements [105]. In the absence of what is deemed a “gold standard,” data consistency was similarly assessed by comparison with paper records [77,83,94], multiple registers [86], or national data [82].

We found that studies’ assessment of uniqueness and timeliness were generally consistent with the DAMA definitions. For uniqueness, studies assessed if records were unexpectedly duplicated, for example, in primary keys [79] or patient names [95]. Similarly, timeliness was consistently assessed as the difference in time between point of data capture versus actual timing of events [74,75,77,81,82,104]. In contrast, some of the DQ concepts reported could not be classified according to the DAMA DQ framework, including simplicity, acceptability, flexibility, stability, usefulness [77], and conformance to a specified data model [90]. These were collectively classified as “other” (3/39, 8%).

### Interventions for Improvement

Studies varied in their approaches to plan and deliver DQ improvement interventions. In total, 20 studies reported using quantitative or qualitative data analysis before planning an intervention. Qualitative analysis involved assessment of clinical workflow inefficiencies through process mapping techniques or staff surveys [74,108]. In contrast, quantitative analysis involved an assessment of DQ with interpretations of possible root causes [95,96].

To understand the types of interventions studied, we identified 5 common themes, including DQ reporting and feedback (24/39, 62%), IT-related or technical solutions (21/39, 54%), training (17/39, 44%), workflow (5/39, 13%), or data cleaning (3/39, 8%). All studies implemented at least 1 intervention with most implementing multiple interventions (23/39, 59%). DQ reporting and feedback involved assessing DQ and sharing curated results with a specific stakeholder with the aim of encouraging improved data capture behavior. These stakeholders included individual clinical staff or managers [87,98,105] or health care institutions as a whole [82,88,90,101,109,110].

Taggart et al [98] implemented structured DQ reports combined with feedback sessions to improve the quality of EHR data in general practice settings. This approach leveraged regular assessments and direct feedback to practice managers to foster ongoing improvements in data recording practices, illustrating a practical application of DQ feedback mechanisms in a real-world health care setting [98]. In contrast, Sinaiko et al [87], studied peer comparison feedback emails in a randomized controlled study to assess its effectiveness on improving cancer stage data completeness, underscoring the importance of control groups in validating the impact of DQ interventions.

We found a range of subthemes under the IT-related or technical-based interventions. These improvements involved either introducing a new electronic data capture system, upgrading an existing one [77,81,93,99,102,103,108,111], enhancing front-end user interfaces [74,78,96], or refining back-end data flow processes. Ahlbrandt et al [96] introduced an intervention focusing on improving the graphical user interface of anesthesia information management systems to enhance the validity of the data captured. By shifting from drop-down lists to radio buttons, rearranging the graphical user interface layout, and limiting user options to a set list, they aimed to reduce invalid data entry by making the interface more intuitive and compliant with data standards. This study exemplifies how interface design can directly influence data validity and highlights the impact of front-end modifications [96].

Technology-based interventions often overlapped with training and workflow changes. Ewing et al [103] implemented a browser-assisted clinical coding software along with training, which, in turn, improved efficiencies in clinical workflows. Other studies mainly targeted workflow inefficiencies [74,85,104,107], with Greiver et al [107] introducing a data entry clerk, whereas Moomba et al [85] shifting data entry responsibility from data entry clerks to frontline clinical staff.

To plan, implement, and assess the impact of these DQ improvement interventions, we found that of the 39 studies, only 5 (13%) used a standardized quality improvement framework or iterative learning cycle, such as PDSA (4/39, 10%) [74,81,84,101] or DMAIC (1/39, 3%) [88]. All 4 PDSA studies completed multiple cycles, ranging from 3 to 8. One study reportedly conducted 421 PDSA cycles across 54 different sites [101]. Larrow et al [74] applied the PDSA method to enhance the timeliness of discharge summaries at a pediatric hospital. The study team initiated their quality improvement project by identifying key barriers through qualitative analysis of staff surveys, leading to the strategic redesign of the EHR structured discharge summary template. Notable enhancements included embedded writing tips and standardized drop-down menus for common diagnoses.

Daniel et al [88] applied the DMAIC methodology to define and assess DQ issues. These authors correlated specific DQ dimensions to possible technical issues, for example, data lacking standardization or valid entries may be caused by “errors from data originators, ETL issues or limitations of the EHR data entry tool (inadequate value set constrains, lack of DQ checks)” [88]. By measuring and analyzing these problems in a structured methodology, the team identified key areas that required targeted interventions, such as use of data standards to enforce data validation rules to the data entry system [88].

Table 5 summarizes the various intervention themes implemented to improve the quality of structured RWD in various health care contexts. It provides insights into common strategies in DQ improvement.

### Reported Outcomes

To understand and compare the outcomes of DQ improvement initiatives, we identified whether studies reported DQ changes that were better, worse, or showed no change over time. Most studies reported improvements in DQ over time (36/39, 92%). This excludes 3 studies due to results being reported as preliminary [88], potential improvements as opposed to actual [92], or without sufficient detail [79]. Of the 36 studies showing improvement, 9 (23%) also report decreases in DQ [96,100,106,109,111], of which another 4 (10%) also report no changes [82,85,98]. These changes were reported at varying levels of granularity. While most studies reported DQ metrics for specific data items, such as validity of surgical procedure codes [96], others aggregated multiple metrics or dimensions into higher level entities, such as “92% reduction in error rate” [112] and mean monthly accuracy for pediatric early warning scores [75].

We also assessed whether statistical tests were used to demonstrate significance of effect and whether studies compared intervention groups with a control group. When determining significance of treatment effect, 22 studies used at least 1 statistical test or method [74-76,78,81-83,87,93,94, 96,98-100,103-107,109,110]. These ranged from chi-square (7/39, 18%) and statistical process control charts (4/39, 10%) to multivariable linear (1/39, 3%) and logistic regression (1/39, 3%). Of these 22 studies, only 1 (5%) study compared the intervention group to a concurrent control group, which reported improvement in completeness of cancer stage data [87].

## Discussion

### Principal Findings

In this paper, we conducted a systematic literature review to understand the current practices in DQ improvement of structured RWD in a health care context. We found substantial heterogeneity in the approaches to definition, assessment, and interventions across the reviewed literature. The range of definitions for DQ concepts, quality improvement methodologies, and reported outcomes have made synthesis and comparison of the results challenging. In the following sections, we explore these 3 points in greater depth.

### DQ Assessment

A key issue in the exploration of DQ is the lack of consensus on theoretical definitions for DQ assessment. Despite the existence of several DQ frameworks, there are no agreed recommendations or guidelines on which frameworks should be used or on how dimensions should be defined, measured, or used to understand real-world issues in data capture, processing, and utility for high-quality RWE generation. This has been demonstrated in a wealth of previous reviews on DQ theory [22,23,25,26,38,48], but to a lesser extent in a quality improvement context. In our review, we found that while some studies did in fact reference theoretical frameworks by Weiskopf and Weng [22] and Kahn et al [38], these account for <15% of all included studies. This indicates a severe lack of uptake of standardized DQ theory in the wider literature and explains the substantial variation and lack of consensus. In turn, the lack of agreement and consistency makes it difficult to harness the true purpose of DQ assessment, which pertains to its ability to identify issues in real-world processes, behaviors, and resources. While some studies demonstrate qualitative correlations between DQ issues and underlying real-world problems [48], we found that only a small minority of studies implemented a quantitative approach to make a similar connection.

As DQ is a complex, multidimensional construct, each dimension serves to identify context-specific issues in the real-world needing remediation. We found that some authors made this correlation either directly or indirectly; for example, data validity is affected by a lack of standardization of front-end user interfaces on electronic data capture forms [96], timeliness of data indicates possible workflow inefficiencies that delays the point of data capture [74,104], duplication highlights redundant data sources [95], inaccurate data underpin lack of training on medical coding standards [103], and inconsistencies between data sources indicate possible capture of inaccurate data [83,86]. This raises 2 important points: the need to assess DQ beyond completeness or missingness and the importance of standardized frameworks. Without these, crucial error-prone processes in complex clinical pathways may go undiagnosed and continue to generate poor-quality data. This is particularly important given the growing demand for and expectations of real-world health care data, the hype in artificial intelligence, and the growing awareness that maintaining patient records is the leading cause of clinician burnout [42,113].

There was limited reporting of the tools or software used for DQ assessment included in this review. Only 2 DQ assessment tools were reported: the WHO DQ assessment toolkit [82,91,94] and the “Open Data Kit” [86]. Neither of these are explained in sufficient depth to discern how they work or their applicability to other environments. Further investigation into the referenced material also lacked sufficient information. Other tools reported were scheduled programmatic scripts using R, SQL, or SAS software for DQ assessment. Some of these methods are considered “automated” solutions for DQ assessment. This indicates a significant gap between the vast range of DQ software available and the practical implementation of these tools for DQ assessment, causal analysis, and improvement. DQ software must be capable of profiling large volumes of structured data, provide both automated and user specified DQ assessment methods, and facilitate meaningful analysis of possible root causes of poor DQ [21,30]. The limited adoption of existing DQ software might suggest a deficiency in technical proficiency, inadequate documentation clarifying its utility or use cases, or a lack of awareness regarding its availability or relevance.

### Quality Improvement Cycles

Another characteristic of the studies included in this review was the limited use of quality improvement frameworks. Only 5 studies [74,81,84,88,101] referenced a quality improvement methodology to plan and implement DQ improvement interventions. This is surprising given the potential benefits these frameworks offer, particularly in fostering systematic, structured, and dynamic approaches to improvement in complex environments.

Quality improvement frameworks, such as PDSA, DMAIC, and TDQM, if implemented robustly, can significantly improve comparability and knowledge sharing between studies, institutions, and organizational teams. This is important given that Siegel et al [100] observed varying improvements across different organizations, stating that systematic and organized quality improvement efforts are needed.

However, the strengths of these frameworks extend beyond iterative learning; they also encourage a deep dive into DQ analysis, helping to unravel the complex relationships between various real-world factors and the root causes of poor DQ. In this way, interventions can be designed in collaboration with the affected stakeholders, that is, frontline clinical staff, to maximize the opportunity for DQ improvement. Knight et al [101] particularly emphasized this point, stating that the quality improvement model, that is, PDSA, “used in this project facilitated the identification and correction of difficulties with the technology of the innovation.” Quality improvement frameworks, such as PDSA, TDQM, or DMAIC, can also be adapted to improve the quality of real-world health care data incorporating DQ-driven quantitative analysis alongside real-world issues that can be identified using the Odigos framework [48].

Despite their strengths, the application of these frameworks is not without challenges. One significant constraint is the need for substantial upfront planning and stakeholder engagement, which can be resource intensive. Furthermore, these frameworks require a culture of continuous improvement and openness to change and adoption of data governance practices, which may not be present in all health care settings. This can limit their applicability and effectiveness. In addition, the lack of consistent application and reporting on the use of these frameworks can make it difficult to evaluate their true effectiveness.

### Outcomes

We sought to investigate the current approaches to DQ assessment and improvement to synthesize and summarize the lessons learnt from these endeavors. In general, studies reported positive changes in DQ through the implementation of multiple interventions. Lemma et al [69] associated the benefits to DQ when interventions such as training, technical innovation, and DQ feedback were combined. The same authors reported that studies that focused only on single interventions did not generate equally positive DQ changes. In contrast, we found that 17 studies focused on a single intervention showing mostly positive results. For example, Sinaiko et al [87] demonstrated the positive impact of peer comparison emails to completion of cancer stage data when compared to a control group. Similarly, studies that demonstrated a combination of improvements, reductions, and no changes in DQ often implemented multiple interventions.

Studies highlighted the importance of close collaboration with clinical users’ needs when implementing digital technologies [101,108,112] and the importance of detailed, personalized feedback on data capture performance provided to data capture end users [87,107,109]. In contrast, Taggart et al [98] found that peer comparison and feedback sessions did not result in better DQ and suggested the need for randomized controlled studies. This raises an important question regarding the need for control groups in DQ improvement studies. In our review, we found only 1 study that compared an intervention group with a concurrent control group, yet most report successful improvement in DQ.

While some studies highlighted the need for controlled environments, we observed that interventions were chosen for reasons other than baseline quantitative or qualitative analysis or analysis of underlying causes. In other words, only a few studies planned an intervention based on a data-driven approach. When comparing outcomes, most studies compared average baselines before and after the intervention, where only 1 study compared the intervention group to a concurrent nonintervention control group. A simultaneous control group for comparison can facilitate analysis of the cause of effect of treatment interventions along with the implementation of multiple interventions, which occurred in most studies. This, combined with the lack of data-driven approaches when planning or designing interventions, indicates a significant gap in robust, standardized DQ improvement methodologies. Therefore, the reported outcomes should be considered with caution.

The methodological rigor in DQ improvement studies often suffers from a lack of randomized controls and consistent statistical methodologies. As discussed before, only 1 reviewed study included a randomized control group to demonstrate significant improvement [87], and just 22 (56%) out of 39 studies applied diverse statistical tests, such as chi-square and logistic regression. This inconsistency in applying robust analytical techniques can introduce biases, misattributing improvements to interventions rather than actual effects. Moreover, the absence of uniform experimental designs across various health care settings undermines the robustness and generalizability of findings.

Incorporating structured methodologies such as PDSA or DMAIC could significantly enhance the methodological rigor of these studies. These frameworks support systematic implementations and evaluations, facilitating the use of control groups and statistical analysis to reliably isolate intervention effects. By adopting such standardized approaches, future research could more effectively ensure the credibility and applicability of the findings, fostering the development of evidence-based interventions suitable for diverse health care environments.

### Future Recommendations

This review highlights the need for standardized and systematic approaches to DQ assessment, analysis, and improvement. This can be addressed in future studies by following quality improvement methodologies, such as the PDSA [114], TDQM [61], or DMAIC [63] iterative learning cycles, and DQ frameworks, such as DAMA [20], Weiskopf and Weng [22], or Kahn et al [38]. Furthermore, understanding the root causes of poor DQ is essential for planning the most appropriate intervention. This intervention should aim to address issues as close to the point of data capture as possible.

The need for standardized DQ assessment is evident. Future research and development should focus on the development and demonstration of DQ tools that are not only grounded in theoretical frameworks, such as those offered by DAMA [20], Weiskopf and Weng [22], or Kahn et al [38], but are also highly accessible and user-friendly. DQ tools should come with comprehensive documentation and practical examples that enable users in making informed decisions about their applicability and relevance in specific health care settings.

As discussed in the Introduction section, DQ tooling currently lack in usability and usefulness [21,29,30]. Ease of use can be overcome by introducing “plug-and-play” functionality that is combined with useful customizable features. This duality can allow users to quickly test and assess the tool’s immediate value and adjust and extend its functionality to fit more complex, specific needs over time. By extension, DQ tools should produce results that are useful for meaningful, in-depth analysis and monitoring of DQ errors.

Current best practices in root cause analysis of poor real-world health care data are unknown but could be facilitated using a framework such as the Odigos framework [48]. Furthermore, understanding causes of poor data can facilitate the design and selection of more relevant interventions needed—an aspect of DQ management that was demonstrated by few articles in this review. Future studies may also wish to compare intervention groups to concurrent control groups and explore 1 intervention at a time instead of multiple. This may help to control for external factors and increase understanding of barriers to high-quality data capture.

### Limitations

This review aimed to summarize the lessons learnt from DQ improvement studies. As an abundance of literature already highlights the substantial variation in terminology for DQ concepts, we used the DAMA DQ framework to standardize the heterogeneity in DQ terms and definitions. In doing so, some DQ concepts could not be classified, potentially affecting the frequency counts of DQ dimensions assessed. Another limitation is that we were unable to perform a comprehensive meta-analysis of the methodological constrains and the effect measures of the reported outcomes. We believe the significant scope of this work warrants future research. This was due to considerable variation in the methods for assessment, analysis, and reporting of DQ metrics and changes over time. This posed significant challenges when attempting to objectively elucidate the effect of treatment interventions.

Moreover, while this review captures a rise in DQ improvement studies, with 31 (79%) out of 39 studies published in the last decade, it also includes 8 studies that were published in or before 2013, potentially missing recent advancements in digital health technologies. In addition, the discovery of 6 additional studies from manual searches indicates the likely exclusion of other relevant work. This is particularly the case due to the lack of consistency in DQ terminology and definitions, which made it difficult to capture all possible variations of DQ terms in the search strategy. Despite these challenges, our review included twice as many studies compared to other related reviews, indicating a thorough coverage within the constraints identified. We believe the significant scope of this work warrants future updates to include emerging trends and methodologies in DQ improvement.

### Conclusions

The reviewed studies demonstrate that approaches to DQ improvement vary in their methodologies, definitions, and reporting of DQ dimensions. In general, studies implemented multiple interventions and reported positive changes in the quality of structured real-world health care data. In addition to “going paperless” initiatives, studies demonstrated the benefits of engagement with frontline clinical end users, provision of personalized DQ feedback, streamlining clinical workflows, and raising awareness of DQ and data standards aimed at improving DQ in health care settings. Despite this, heterogeneity is a major limitation among DQ literature in general, and we recommend that studies refer to standardized frameworks, such as PDSA cycles for quality improvement and the DAMA DQ framework for assessing DQ dimensions. This would lead to greater consistency and comparison in the reported outcomes.

## Appendix

Multimedia Appendix 1 PRISMA (Preferred Reporting Items for Systematic Reviews and Meta-Analyses) checklist.

Multimedia Appendix 2 Search strategy and results.

## Abbreviations

DAMA: Data Management Association
DMAIC: define-measure-analyze-improve-control
DQ: data quality
EHR: electronic health record
EMA: European Medicines Agency
EMR: electronic medical record
FDA: Food and Drug Administration
HIS: hospital information system
HL7: Health Language 7
ICD: International Classification of Diseases
ICPM: International Classification of Procedures in Medicine
LHS: learning health care system
MeSH: Medical Subject Headings
MHRA: Medicines and Healthcare Products Regulatory Agency
NICE: National Institute for Health and Care Excellence
OPS: Operationen und Prozedurenschlüssel
PACS: picture archiving and communication system
PDQI-9: Physician Documentation Quality Instrument
PDQI-9: Physician Documentation Quality Instrument
PDSA: plan-do-study-act
PRISMA: Preferred Reporting Items for Systematic Reviews and Meta-Analyses
RWD: real-world data
RWE: real-world evidence
SQL: structured query language
TDQM: total data quality management
WHO: World Health Organization

## Data Availability

All data generated or analyzed during this study are included in this manuscript and its multimedia appendices.
