# Supplementary material for: Data Quality–Driven Improvement in Health Care: Systematic Literature Review
Source: J Med Internet Res. 2024 Aug 22;26:e57615. doi: 10.2196/57615 (PMC11377907; doi:10.2196/57615)
Supplement: Multimedia Appendix 2 [file jmir_v26i1e57615_app2.docx]

## Multimedia Appendix 2

Table S1: PICOC search strategy terms.* = include synonyms of these words.

| Criterion | Description | Search Terms | MeSH Terms or Keywords |
| --- | --- | --- | --- |
|  |  |  |  |
| Population | DQ of EHR systems. | *Data Accuracy/ or Data Accuracy.mp. | “Data Accuracy” |
|  |  | (*Electronic Health Records/ or Electronic Health Records.mp.) or (*Medical Records Systems, Computerized/ or Medical Records Systems, Computerized.mp.) | “EHR" or “EMR” |
| Intervention | Tools, methods, and interventions used to assess DQ. | N/A | N/A |
| Comparison | N/A | N/A | N/A |
| Outcomes | DQ improvement or changes over time. | *Quality Improvement/ or Quality Improvement.mp. | “Quality Improvement” |
| Context | Structured EHR data, healthcare, and empirical publications. |  |  |

Table S2: Search strategy and results using the Ovid MEDLINE database from 1945 to 17 July 2023.

| ID | Query | Results |
| --- | --- | --- |
| 1 | *Data Accuracy/ or Data Accuracy.mp. or DQ.mp. | 14,840 |
| 2 | *Quality Improvement/ or Quality Improvement.mp. | 70,649 |
| 3 | *Electronic Health Records/ or Electronic Health Records.mp. or (*Medical Records Systems, Computerized/ or Medical Records Systems, Computerized.mp.) | 55,155 |
| 4 | 1 and 2 and 3 | 104 |

Table S3: Search strategy and results using the Pubmed database from 1945 to 17 July 2023.

| ID | Query | Results |
| --- | --- | --- |
| 1 | ("data accuracy"[MeSH Terms] OR ("data"[All Fields] AND "accuracy"[All Fields]) OR "data accuracy"[All Fields] OR "DQ"[All Fields]) | 209,686 |
| 2 | "quality improvement"[MeSH Terms] OR ("quality"[All Fields] AND "improvement"[All Fields]) OR "quality improvement"[All Fields] | 196,676 |
| 3 | "electronic health records"[MeSH Terms] OR ("electronic"[All Fields] AND "health"[All Fields] AND "records"[All Fields]) OR "electronic health records"[All Fields] | 66,184 |
| 4 | ("data accuracy"[MeSH Terms] OR ("data"[All Fields] AND "accuracy"[All Fields]) OR "data accuracy"[All Fields] OR "DQ"[All Fields]) AND ("quality improvement"[MeSH Terms] OR ("quality"[All Fields] AND "improvement"[All Fields]) OR "quality improvement"[All Fields]) AND ("electronic health records"[MeSH Terms] OR ("electronic"[All Fields] AND "health"[All Fields] AND "records"[All Fields]) OR "electronic health records"[All Fields]) | 416 |
